# Study on the Development of Antiviral Spandex Fabric Coated with Poly(Hexamethylene Biguanide) Hydrochloride (PHMB)

**DOI:** 10.3390/polym13132122

**Published:** 2021-06-28

**Authors:** Wen-Yi Wang, Sui-Lung Yim, Chun-Ho Wong, Chi-Wai Kan

**Affiliations:** 1 Institute of Textiles and Clothing, The Hong Kong Polytechnic University, Hong Kong 999077, China; tcwang@polyu.edu.hk; 2 Avalon SteriTech Limited, Shatin, New Territories, Hong Kong 999077, China; rogeryim@avalonbiomedical.com (S.-L.Y.); chwong@avalonbiomedical.com (C.-H.W.)

**Keywords:** PHMB, antiviral clothing, coronavirus, protective spandex clothing

## Abstract

The spread of COVID-19 has brought about huge losses around the world. This study aims to investigate the applicability of PHMB used for developing antiviral spandex clothing against coronavirus. PHMB was qualitatively determined on the surface of spandex fabrics by using BPB. The antiviral analysis shows that the PHMB-treated spandex fabric can kill 99% of the coronavirus within 2 h of contact, which suggests that the spandex fabric treated with PHMB could be used for developing antiviral clothing against coronaviruses for containing the transmission of COVID-19 in high-risk places. Furthermore, PHMB-treated spandex fabrics were shown excellent antibacterial activity against gram-positive *S. aureus* and gram-negative *K. pneumoniae*. The hand feel properties of Spandex fabric were not significantly affected by the PHMB coating in addition to the wrinkle recovery, which was obviously improved after PHMB coating.

## 1. Introduction

Spandex fiber is a long-chain synthetic polymeric fiber which was first invented at DuPont’s laboratory in 1959 [1]. It is reported that spandex fabric can be stretched up to five to eight times its normal size and then recover to its original shape [2]. Spandex fabric (Lycra^®^, spandex, and elastane are mostly synonyms) is gaining popularity in making stretchable clothing in recent years due to its extraordinary merits such as exceptional elasticity, durability, and light weight [3]. In addition to this, spandex fabric is supple and more adaptable, which makes spandex widely used in garments where both comfort and fit are required such as hosiery, swimsuits, exercise wear, surgical hose, undergarments, gloves, cycling shorts, wrestling suits, and rowing suits [4,5,6]. 

There are four methods available for fabricating spandex fibers, i.e., melt extrusion, reaction spinning, solution dry spinning, and solution wet spinning, among which solution dry spinning is the most used method for fabricating spandex, with nearly 95 percent of the world’s spandex being manufactured by this method [2]. Briefly, the first step included in this method is the production of a prepolymer by mixing macroglycol (polyester, polyether, polycarbonate, polycaprolactone, or a combination of these chemicals) with a diisocyanate monomer. The prepolymer is then reacted with diamine acid to extend the chain of the prepolymer. Afterwards, this solution is diluted to make it easier to handle before extrusion [7]. Through heating, twisting, finishing, and weaving, the elastic and durable spandex fabrics are finally produced. The stretching characteristic of spandex is ascribed to the hydroxyl groups on both ends of the macroglycol, while the strength results from the shorter chain polymer isocyanate group [1]. Though spandex fabric has lots of advantages as mentioned above, it also suffers from some disadvantages with its poor breathability and hygroscopicity. These problems can be addressed by blending cotton or other breathable materials with spandex [8].

Textile fibers play a significant role in the transmission of infectious diseases because of the vulnerability to microbial attack. The outbreak of COVID-19 around the world has gone on for one and a half years and has already resulted in huge losses [9]. It has been reported that COVID-19 could survive for 48 h on cloth [10], which makes it indispensable to develop antiviral fabrics with potential application in protective clothing. In order to achieve antiviral activity, a wide variety of chemicals have been applied to textile products such as triclosan via finishing technology [11]. Apart from the broad-spectrum bactericidal properties, one of the major problems for these treated antiviral fabrics is the durability against wear and household washing. 

**Figure 1:**
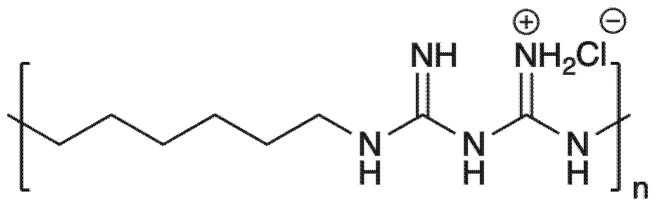
Chemical structure of PHMB.

Poly(hexamethylene biguanide) hydrochloride (PHMB) is a well-known commercial bactericide that has been widely used in a variety of areas such as the food industry and recreational water because of its low toxicity and broad-spectrum bactericidal properties [12,13]. PHMB is composed of repeating biguanidine units spaced by hydrophobic hexamethylen hydrocarbon chains (Figure 1). The antibacterial mechanism of PHMB is highly dependent on the cationic biguanide moieties that can interact with the negatively charged phosphate head groups of bacterial cell membrane and finally cause cell death [14,15]. In the textile industry, PHMB is generally used through the processes of padding, spraying, conventional exhaust method or pad–dry–cure [16]. PHMB shows strong affinity to cellulosic fibers due to electrostatic interaction and hydrogen bonding and the adsorption follows typical Langmuir isotherms at lower concentrations [13]. Therefore, the fabrics treated with PHMB display excellent durability against household laundering [17]. Moreover, PHMB has modest virucidal activity against non-enveloped viruses such as HIV-1 [18]. The mechanism of action of PHMB-based biocides is to interact with the viral capsid and to lead to virus death [19]. Hence, the present study aims to develop an antiviral fabric against COVID-19 with potential application for protective clothing by using PHMB.

## 2. Materials and Methods

Spandex-knitted fabric (cotton 92%/spandex 8%) with weight 180 g/cm^2^ was used in this study. Poly(hexamethylene biguanide) hydrochloride (PHMB) as a 20% *w*/*v* aqueous solution, PEG400 (400 g/mol in average), polyurethane binder (20% *w*/*v* aqueous solution), and bromophenol blue (BPB) sodium were supplied by Sigma-Aldrich (St. Louis, MO, USA).

### 2.1. PHMB Coating Treatment

The finishing recipe was prepared by adding 10% (*w*/*v*) PHMB, 5% (*w*/*v*) PEG400, and 8% (*w*/*v*) binder into deionized water. The process of pad–dry–cure was used to treat the fabric samples. The fabric sample was firstly padded with the finishing solution with a wet pickup of 80%. Afterwards, the sample was dried in an oven at 90 °C for 5 min and then cured at 130 °C for 45 s.

### 2.2. Qualitative Analysis of PHMB

The presence of PHMB on the fabric was qualitatively determined by the anionic dye BPB, which can be complexed with PHMB with the formation of a blue stable complex [16]. The present study utilizes BPB to qualitatively determine the presence of PHMB coated on the fabrics.

### 2.3. Antiviral Activity

The antiviral activity of the fabric samples treated with PHMB was tested in accordance with ISO 18184:2019. The antiviral fabrics (20 × 20 mm) were inoculated with 200 μL of virus at a concentration of 105 and left for 2 h at 25 °C. After contacting for 2 h, 20 mL of wash-out solution in the vial containers was added with agitation by Vortex mixer for 5 s and 5 times to wash out the virus from the specimens. TCID50 (Median Tissue Culture Infectious Dose) was calculated following the appropriate incubation time. The antiviral activity value was then calculated by comparison of the antiviral test recover from the control fabric according to Equation (1) as follows:

*M* = lg(*V_a_*) *−* lg(*V_b_*) (1)

where *M* is the antiviral activity value, lg(*V_a_*) is the common logarithm average infectivity titer after 2 h of contact (PFU/vial) with the reference specimen, and lg(*V_b_*) is the common logarithm average infectivity titer after 2 h of contact with the antiviral spandex fabric.

### 2.4. Antibacterial Property

The antibacterial property of treated samples was qualitatively investigated against gram-positive *S. aureus* and gram-negative *K. pneumoniae* according to AATCC TM 147-2011, and the quantitative determination was performed in accordance with AATCC TM100-2019. The bacteria were grown in nutrient agar at 37 ± 2 °C for 24 h. Fabric samples were cut into 20 × 20 mm pieces for the antibacterial test. The zone of inhibition around the samples were photographed at the end of the incubation period. The average width of inhibition zone along a streak on either side of the sample was calculated by Equation (2) as follows:

*W* = (*T* − *D*)/2 (2)

where *W* = width of clear zone of inhibition in mm; *T* = total diameter of test specimen and clear zone in mm; and *D* = diameter of the test specimen in mm. The Inhibition percentage of PHMB-coated spandex samples were calculated as per Equation (3):

*Inhibition* % = [(*A* − *B*)/*A*] × 100% (3)

where *A* = CFU/mL for sample at 0 h and *B* = CFU/mL for sample after 24 h.

### 2.5. Hand Feel Evaluation

The hand feel properties of Spandex after PHMB coating, i.e., resilience, softness, smoothness, and wrinkle recovery, were evaluated by using a PhabrOmeter instrument (Nu Cybertec, Inc., Davis, CA, USA) according to AATCC Test Method 202-2014. 

## 3. Results and Discussion

### 3.1. Qualitative Analysis of PHMB Coated on the Surface of Spandex Fabric

**Figure 2:**
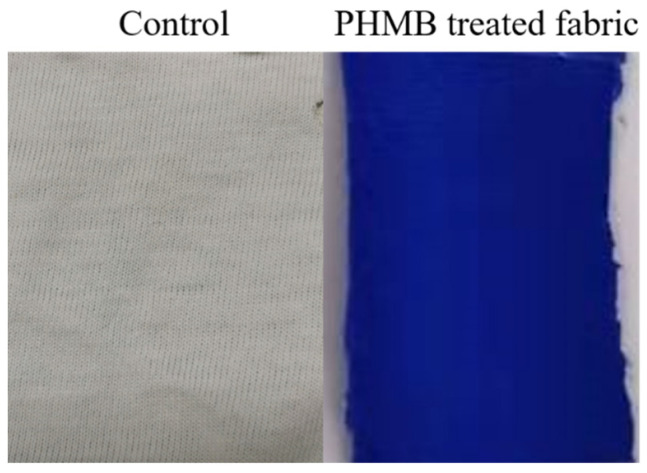
Images of the untreated fabric and PHMB treated fabric sample.

Spandex fabric is distinguished for its remarkable elasticity, which is desired in many applications such as form-fitting garments, sportswear, and glove fabric. The present study aims to impart spandex fabric with antiviral activity against coronavirus by using PHMB. Figure 2 demonstrates that the spandex fabric treated with PHMB shows a distinct blue shade, as compared to the control sample, which suggests the presence of the PHMB coating on the surface of the spandex fabric sample. 

### 3.2. Antiviral Activity of PHMB Coated Spandex Fabric

Then, the antiviral activity of the PHMB-treated spandex fabric against Feline coronavirus, a surrogate virus of SARS-CoV-2, was studied and shown in Table 1. It can be clearly observed that the PHMB-treated spandex fabric had strong antiviral behavior against Feline coronavirus after 2 h of contact, with up to 99% viral inactivation. The antiviral activity value is 2.21. SARS-CoV-2 can survive for 48 h on cloth according to report [9]. This demonstrates that the PHMB-treated spandex fabrics could effectively contain the transmission of coronavirus. The action mechanism could be due to the interaction of PHMB with the viral capsid, leading to virus death [19]. Considering the susceptibility of COVID-19 to standard disinfection methods [9], it is believed that PHMB-treated spandex fabrics could play a role in containing the transmission of COVID-19, particularly in high-risk places. 

### 3.3. Antibacterial Properties of PHMB-Coated Spandex Fabric 

**Figure 3:**
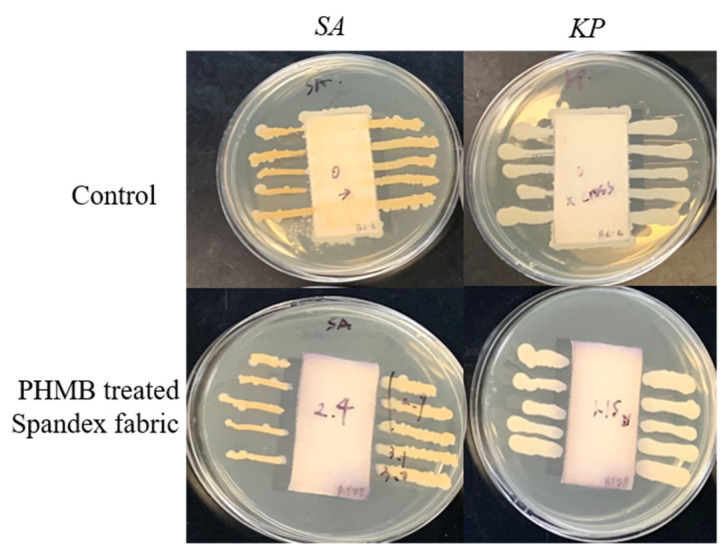
Images of the qualitative analysis for antibacterial activities of PHMB-coated fabric samples against *S. aureus* (*SA*) and *K. pneumoniae* (*KP*).

Next, the antibacterial activity of spandex fabrics after PHMB treatment was evaluated both qualitatively and quantitatively. Figure 3 displays the images of control sample and PHMB treated spandex fabric in the inhibition of *S. aureus* and *K. pneumoniae*. The inhibition zone and inhibition percentage against *S. aureus* and *K. pneumoniae* were shown in Table 2. Clearly, the bacteria passing throughout the fabric samples reveals that the control sample does not have any antibacterial properties. By contrast, after treatment with PHMB, the spandex fabric shows strong antibacterial behaviour, particularly for the gram-positive *S. aureus* with the inhibition zone of 2.40 mm, which is far greater than that for the gram-negative *K. pneumoniae*. The reason for this could be due to the fact that *S. aureus* is more vulnerable to the antibacterial agent PHMB than *K. pneumoniae*. The quantitative analysis further showed that the spandex fabric after PHMB treatment has 100% inhibitory action against both *S. aureus* and *K. pneumoniae*. The mechanism of action could be explained by the fact that PHMB molecules can disrupt the microbial membrane and selectively condenses the chromosomes, causing microbe death [14].

### 3.4. Hand Feel Analysis

The PHMB coating may affect the hand feel properties of spandex fabric. Hence, the hand feel of PHMB treated spandex was evaluated and shown in Table 3. Clearly, after coating with PHMB, both resilience and softness of spandex fabric have a negligible decrease, whereas there is a slight increase in the smoothness. These results show that the PHMB coating slightly affects the hand feel properties of spandex. However, the wrinkle recovery saw a significant increase from 65.2% to 73.16% in comparison to the control sample. This suggests that the PHMB coating enhances the wrinkle recovery of spandex.

## 4. Conclusions

**Figure 4:**
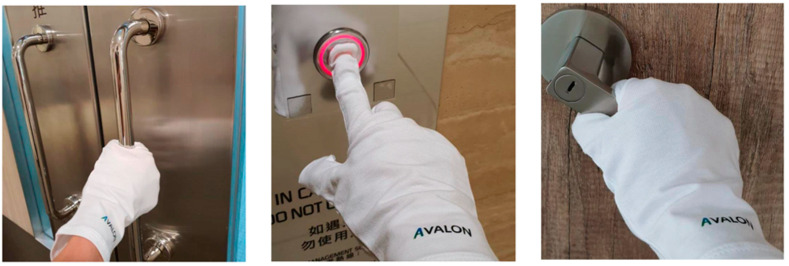
Spandex-based antiviral gloves on various daily life scenarios.

The antiviral activity of the PHMB-treated spandex fabric was investigated in the present study against Feline coronavirus. BPB testing shows that PHMB was successfully coated on the surface of fabric. The antiviral analysis shows that the PHMB-treated spandex fabric killed 99% of the coronavirus within 2 h contact, which suggests that the spandex fabric treated with PHMB could be used for developing antiviral clothing (e.g., gloves in Figure 4) against coronaviruses for containing the transmission of COVID-19 from high-risk surfaces. Moreover, both qualitative and quantitative antibacterial analyses demonstrated that the spandex fabric has excellent antibacterial activity after treatment with PHBM, particularly for the gram-positive *S. aureus*. Hand feel analysis showed that PHMB coating does not visibly affect the hand feel properties such as resilience, softness, and smoothness, but can obviously improve the wrinkle recovery of Spandex fabric.

## Figures and Tables

**Table 1:** Antiviral activity values of the control and PHMB treated fabric samples.

Sample	Logarithm Average of Infectivity Titer (PFU/vial)		Antiviral Activity	Percentage/%
Control	Immediately after inoculation	5.71	N/A	N/A
	After contacting for 2 h	5.24	0.47	66.29
PHMB-treated spandex fabric	After contacting for 2 h	3.50	2.21	99.38

**Table 2:** Inhibition zone and inhibition percentage of PHMB-treated spandex fabrics against *S. aureus* (*SA*) and *K. pneumoniae* (*KP*).

Sample	Inhibition Zone/mm		Inhibition/%	
	*SA*	*KP*	*SA*	*KP*
Control	0	0	N/A	N/A
PHMB-treated spandex fabric	2.40	1.15	100	100

**Table 3:** Hand feel properties of PHMB-coated spandex fabric.

Sample	Resilience	Softness	Smoothness	Wrinkle Recovery/%
Control	41.85	88.58	54.44	65.2
PHMB-treated spandex fabric	39.69	88.37	55.56	73.16

## Data Availability

The data presented in this study are available on request from the corresponding author.
